# “*Each one of us did the best we could for the community*,* while also supporting each other”*: community residents’ perspectives on community health worker (CHW) response during the COVID-19 pandemic - a community science worker-led qualitative study

**DOI:** 10.1186/s12889-025-22497-7

**Published:** 2025-04-04

**Authors:** Melina Michelen, Beatriz Lopez Galeana, Salvador Zárate, Sora Park Tanjasiri, Lindsay Donaldson, Patricia J. Cantero, Noraima Chirinos, Rocio Salazar, Mary Anne Foo, Samantha Peralta, Pilar Lara de Cortez, Guadalupe Capistran, John Billimek, Alana M.W. LeBrón

**Affiliations:** 1 Joe C. Wen School of Population & Public Health, University of California, Irvine, CA USA; 2 Department of Anthropology, School of Social Sciences, University of California, Irvine, CA USA; 3 Claremont Graduate University, Claremont, CA 91711 USA; 4 Latino Health Access, Santa Ana, CA USA; 5 Orange County Asian and Pacific Islander Community Alliance, Garden Grove, CA USA; 6 Radiate Consulting, Santa Ana, CA USA; 7 Department of Family Medicine, University of California, Irvine, CA USA; 8 Department of Chicano/Latino Studies, School of Social Sciences, University of California, Irvine, CA USA

**Keywords:** Community health worker, Mutual aid, COVID-19 pandemic, Immigrant health, Latino, Latinx, Vietnamese, Korean

## Abstract

**Background:**

The COVID-19 pandemic significantly disrupted the health and social wellbeing of the United States population, disproportionately affecting low-income, immigrant communities of color. In Orange County, California, community health workers (CHWs) were essential to addressing multilevel community needs among impacted communities. However, little is known about how communities and CHWs responded to meet their needs amid pressing challenges.

**Methods:**

CHWs completed a popular education qualitative methods program under a Community Science Worker (CSW) model to design and facilitate four semi-structured focus groups and three interviews with 32 residents in Orange County, California, to understand their pandemic experiences and interactions with CHWs. Sessions were recorded, transcribed, and analyzed using an adapted flexible coding approach to derive data-driven themes.

**Results:**

Residents described how they supported one another, advocated for their communities, and fostered livelihood and resilience. Four main themes detail the community’s response: (1) facing a chain of interconnected challenges; (2) connecting with CHWs and accessing the services they facilitated; (3) fostering a community of care, a mutuality often inspired by interactions with CHWs; and (4) reinforcing foundations through a whole-of-community approach, including strengthening social policies.

**Conclusions:**

Engaging community members is crucial for comprehensively understanding the CHW model. Given the enormous ongoing community challenges post-pandemic, these findings call for increased CHW presence, additional support and resources for health and socioeconomic needs, and improved information dissemination to bolster community resilience. Findings center mutual aid, emphasizing the importance of supporting communities in this crucial work. Additionally, engaging with residents who CHWs supported is vital for understanding the full impact of CHW models.

## Introduction

The COVID-19 pandemic (henceforth, “the pandemic”) starkly exposed and deepened the structural power imbalances and racial capitalist exploitation that perpetuate health inequities among low-income communities and communities of color in the United States [1–3]. These systems of injustices led to pronounced inequities in COVID-19 exposure, testing, infection rates, health outcomes, and mortality among historically and contemporarily marginalized and systematically excluded populations [4–6]. While the pandemic was declared over in May 2023 [7], communities continue to face the compounding effects of worsened socioeconomic inequities, hindering their ability to rebuild and prepare for future public health crises.

### Community-based pandemic response

Community Health Workers (CHWs) and Community-Based Organizations (CBOs) have been instrumental in leading the COVID-19 response, using their experiential knowledge to address the failures of established systems in serving low-income communities and communities of color [8–13]. As trusted leaders and community members with shared experiences of oppression, CHWs work closely with their communities to address the social and economic injustices impacting health by centering community member expertise and experiences [14, 15]. CHWs are uniquely positioned to improve health equity by delivering physical and mental health services, navigating residents through complex processes, addressing structural barriers such as the need for structurally and culturally responsive health information, and fostering civic engagement to advocate for systemic changes that improve community wellbeing [15]. During the pandemic, CHWs addressed immediate response and recovery challenges while also contributing to long-term community health and whole-person care [16]. The CHW response embodies mutual aid practices, where communities facing common challenges unite to understand the conditions of inequity together while supporting each other in a practice grounded on reciprocity and solidarity [17, 18].

### CHW engagement and community perspective in research

Although CHW models highlight the value of CHWs’ experiential knowledge and navigation skills, the literature on CHW models often lacks direct engagement of CHWs in the research process. There is a recognized need to increase CHWs’ involvement in research, as they bring valuable practice-based knowledge to community-academic collaborations [19–21]. However, gaps remain; a recent scoping review found that only 23 of 130 articles involved CHWs in five or more phases of intervention research, with limited participation in identifying research questions (10.8%), data analysis (2.3%), and dissemination (10.8%) [22].

Further, many studies focus on CHW model outcome assessments, with few specifically examining the experiences of communities who work with CHWs and wider impacts of CHW models [23, 24]. There is a critical need to better understand community-led strategies employed during the pandemic and the role of CHWs in strengthening community-led efforts. As the pandemic continues to disproportionately affect low-income communities and communities of color, it is increasingly important to support community-driven solutions to address unmet needs and systemic inequities.

#### Research questions

This study aims to investigate community approaches in how Latiné (a gender-inclusive term to refer to people of Latin American origin or descent) [16] and Asian residents navigated the challenges posed or worsened by the pandemic and the role of CHWs in community-based assistance. Specifically, we addressed these questions by developing and applying a Community Science Worker (CSW) model, wherein CHWs guide the research questions, data collection, analysis, and dissemination efforts, offering direct insights into community perspectives. To foster equitable recovery efforts, we must sustain and support these crucial, impactful community efforts without further burdening already impacted communities.

## Methods

### Setting & design

This qualitative study uses data from a subset of semi-structured in-depth focus groups and interviews conducted as part of the Community Activation to TrAnsform Local sYSTems (CATALYST) study [25]. CATALYST followed a phenomenological research design in developing the data collection process and guiding the analytic process and was informed by community partners’ expertise and two robust conceptual models, the National Institute on Minority Health and Health Disparities (NIMHD) Research Framework and the Multisystemic Promotores/Community Health Worker Model [15, 26]. Study protocols were approved by the institutional review board in 2020 (UCI IRB #1272).

The study took place in Orange County, California, the sixth largest county in the U.S. [27], which exhibited heightened racial and ethnic inequities in COVID-19 outcomes, reflecting national trends [28–31]. The pandemic exacerbated long-standing exclusions of immigrant communities—especially undocumented residents—from social, economic, and health resources [32]. Many mixed-status families reside in the county, where some members are undocumented while others hold authorized immigration status [33]. During this time, exclusionary immigration policies and discussions around public charge heightened fears about accessing public services, further limiting access to essential resources [32, 34].

### Author positionality

This diverse multilingual community-academic partnership is deeply committed to social justice, health equity, and participatory research and action and has collaborated for several years, fostering strong, trust-based relationships. The primary languages spoken by the authors are English and Spanish. The team includes numerous Latiné, Asian American, and Pacific Islander (AAPI) scholars and community leaders and members, with many identifying as women. Research processes and discussions were guided by community members and leaders with lived experiences of injustices and inequities in Orange County, ensuring that their voices and perspectives are central to the work. Several co-authors brought their expertise in qualitative research, enriching the research with their insights.

### Community science worker (CSW) program development

Our partnership recognized that the structural space between academic researchers and community residents disproportionately affected by the pandemic could hinder rapport-building with participants, potentially limiting the depth of insights we could derive. Accordingly, we recognize that CHWs are well-positioned to guide the research process, contributing to the development of the CSW model [25]. The CSW model is grounded in popular education, which emphasizes group learning as a way to uncover collective strength and foster community-driven solutions by challenging traditional power dynamics and the ‘expert paradigm’ [35]. Through the CSW model, CSWs combined their experiential knowledge and skills with their training to drive this process, as described in Table 1 and the sections that follow.

**Table 1:** Community science worker research co-design program components

Component	Description
Nominations	Community partners nominated CHWs within their organization or network who had several years of experience as a CHW, expressed interest in learning about and/or applying research and evaluation skills, and were available to support and participate in this intensive research effort in addition to their CHW responsibilities. The research team invited the CHWs to join the project as CSWs (*n* = 12).
Research Ethics	CSWs completed an IRB-approved research ethics certification designed for use by team members within and outside of academic institutions and available in several languages [36].
Research Co-design	CSWs completed 8 bilingual (English/Spanish) in-person and remote research design sessions to: ● Determine what questions to ask ● Select participants and recruitment criteria ● Develop methods and tools for a sking questions: Learn the strengths of various qualitative research methods, including photovoice, focus group discussions, and arts-based expression; identify most appropriate methods; and determine how to effectively apply these methods.
Data Collection	CSWs led data collection using qualitative methods that were best suited to their communities, employing facilitation guides and strategies tailored to each group.
Analysis & Interpretation	CSWs played a central role in analyzing the data and interpreting the findings through a series of sessions, drawing on their community knowledge to provide contextually grounded insights.

### Participant recruitment

CSWs recruited participants via purposive sampling, ​​leveraging the networks of the coauthors, wherein CSWs nominated adults who had been supported by other CHWs in their organization in Orange County during the pandemic. Eligible participants were contacted by CSWs via CSW-co-designed flyers or phone calls and invited to contact the study team if interested in participating. The study team screened and scheduled participants. Considering participant time constraints and that studies have shown 80% of themes are achieved within two to three focus groups, and 90% within three to six [37, 38], a sample size of four focus groups (5–10 participants each) and three interviews was deemed appropriate for this analysis.

### Data collection

CSWs designed and piloted discussion guides [25] for data collection focused on residents’ experiences during the pandemic (see appendix 4 of the protocol). The guides were professionally translated by consultants or team members who are native speakers, then reviewed and piloted by community partners and CHWs.

Interviews and focus groups were conducted in-person at locations comfortable for participants (community spaces, clinics, etc.). Discussions were semi-structured to foster open dialogue and interactions among participants and between participants and facilitators. Each data collection event was scheduled in-person for 2 h and included a meal, verbal informed consent from all participants, a demographics survey, and a semi-structured in-depth discussion. We obtained IRB approval for verbal informed consent due to the nature of our study and the priorities and challenges (e.g., reducing data identifiability) identified by community partners. In accordance with IRB recommendations, we obtained informed consent from each participant, after sharing the study information sheet and an accompanying video to remind participants that their involvement was voluntary, they could stop at any time, and that their data would be safeguarded. A woman-identifying CSW from the same community as participants facilitated the discussion in participants’ preferred language (Spanish, Vietnamese, and Korean). Another CSW supported notetaking. All discussions were audio/video recorded. Participants received a $50 incentive as a token of appreciation for their participation in the discussion.

### Data analysis

Recorded discussions were professionally transcribed, translated into English, and entered into Atlas.ti (Version 23.2.1) qualitative data analysis software. The analysis process followed an adapted flexible coding approach [39], resulting in key themes and categories in each domain of inquiry. This approach began with indexing, or a broad data categorization guided by the discussion questions developed by CSWs. During this process, analytical memos helped document the concepts arising from the data review. CSWs reviewed the analytical memos and indexed data to identify priority research questions. Then, CSWs and academic researchers discussed key concepts and differences they were observing to develop a focused analytical codebook, with inductive and deductive codes across three broad categories of impact, support, and recommendations.

The data was then coded through paired coding by two independent coders. Coders met regularly to discuss and reconcile inconsistencies in coding and assess the reliability of the coding process, using intercoder agreement (ICA). ICA was measured using Krippendorff’s α to calculate the reliability coefficient, with a score above 0.80 indicating high reliability. Writing memos during this phase facilitated data interpretation and refinement. Additionally, CSWs reviewed the memos, assisted in interpreting the initial findings, and guided the analysis. Conceptual relationships and code frequency were reviewed using matrices from Atlas.ti. Discussions with CSWs provided deeper texture and context for findings. We maintained a system for recording codebook modification. Saturation was deemed achieved when no new codes were documented and modifications ceased. Additionally, we compared insights from codes across transcripts, considering demographic differences, to confirm no new patterns were observed.

## Results

Participant demographics (*n* = 32) are shown in Table 2. The final sample was primarily female (88%) and 50 years of age or older (66%). The majority identified as Latiné (75%) and most resided in Santa Ana (63%). Other cities represented included Anaheim, Buena Park, Fullerton, lake Forest, Stanton, and Wesminster. Interviews and focus groups resulted in 364 min of recording, with the median duration being 54 (20–84) minutes.

**Table 2:** Participant demographics

Characteristics		*n* (%) (*N* = 32)
Sex	Female	28 (88%)
	Male	4 (13%)
Age	18–40	5 (16%)
	40–50	6 (19%)
	50–60	13 (41%)
	60–90	8 (25%)
Ethnicity/Race	Latiné	24 (75%)
	Vietnamese	5 (16%)
	Korean	3 (9%)
City of residence	Santa Ana	20 (63%)
	Another Orange County City	9 (3%)
	Not Reported	3 (9%)

**Fig. 1:**
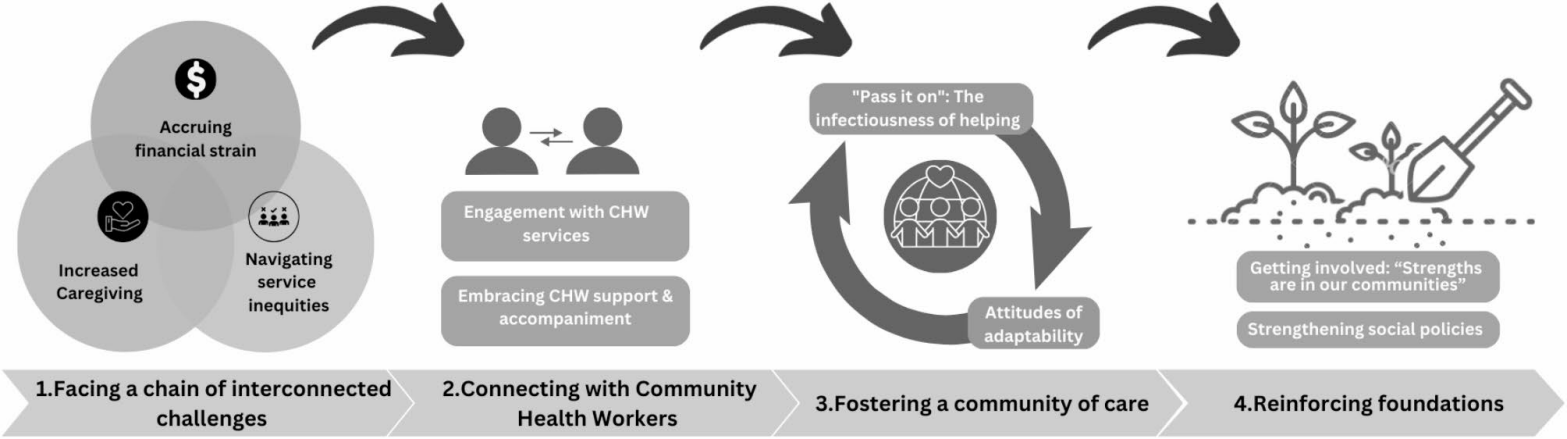
Themes and categories of resident experiences and responses

The following sections detail the findings, illuminating that marginalized communities were left to navigate increasingly complex and interconnected challenges during the pandemic. These difficulties necessitated that community members engage with CHWs leading to a network of mutual support. Furthermore, communities expressed a desire to be involved in addressing systemic gaps and strengthening social policies. Themes and categories are presented in Fig. 1 and described narratively with supporting quotes in the sections that follow.

### Theme 1: facing a chain of interconnected challenges

Residents’ narratives vividly illustrate the multitude of challenges they faced, including financial strain, increased caregiving responsibilities, and exclusion from essential services, often concurrently and unexpectedly. These interconnected issues compounded and intensified their hardships, generating feelings of fear and uncertainty, often leaving residents unsure of where to seek help or how to navigate their situations. Some described “not knowing which way to walk or where to hold on” and being “left in a dead-end circle”.

#### Accruing financial strain

Many residents described financial strain and income loss during the pandemic. They recounted how their low-wage jobs linked with service and informal economies—including in housekeeping, childcare, and gardening—lacked a safety network, leaving them particularly vulnerable to economic instability. One participant shared their experience, explaining that many community members, already living day-to-day with minimal or no savings, faced challenges when terminated without notice or compensation: “I’m a woman who lives day by day, which I never imagined. I was like, ‘Well, I have my job. It’s never going to end after ten, 15 years, working with people.’ And to be told, ‘You know what? I don’t need you anymore, because there’s this and that going on.’ Without saying, ‘Thanks,’ or ‘Do you need anything?’[…] I was really affected by that. Because, well, I was like a total baby, not knowing which way to walk or where to hold on.” **(Resident Focus Group 4**,** Latiné).**

Many community members closely linked financial instability with housing and food insecurity. One participant poignantly stated: “What are we going to do? The job’s gone. We’ve got no food. Where are we going to run to?” **(Resident Focus Group 4**,** Latiné).** In advocating for financial assistance for recovery, a participant shared the challenge families faced when moving into smaller places: “[…] many people lost their jobs, they moved to smaller places, they are living in one room, a family with three children, that’s six children in one room.” **(Resident Focus Group 3**,** Latiné).** Another described the measures taken to pay rent, sacrificing longer-term financial security to meet unprecedented short-term economic needs: “I had to cancel my pension plan. So it was a difficult situation.” **(Resident Interview 3**,** Korean)**. One participant described how a lack of information affected housing, even with an eviction moratorium in place: “No one in COVID should’ve been evicted, but the problem is that there’s not much word of mouth happening. And so, not everyone knew about it. But there shouldn’t have been any evictions.” **(Resident Focus Group 4**,** Latiné).**

Similarly, participants highlighted the interconnectedness of financial strain with increased healthcare needs during the pandemic due to mental and physical illness. Many community members echoed concerns about being unable to earn income if they were sick or had to care for their families while sick. One participant shared their husband had complications due to COVID-19, which affected them financially: “I couldn’t work much. And I was taking SSI [Supplemental Security income] because my husband was sick. However, it was materially difficult because I couldn’t make more money.” **(Resident Interview 3**,** Korean).** Another participant discussed difficulties with mental health services, noting the financial burden: “Mental health is something we have to work a lot on […], and it’s extremely expensive” **(Resident Focus Group 4**,** Latiné).**

#### Increased caregiving

Participants discussed the increased caregiving responsibilities that arose, which were often gendered. Highlighting the interconnectedness of these issues, increased caregiving often worsened residents’ financial strain, which in turn intensified related challenges including housing, food instability, and emotional impacts. Increased caregiving was often the result of family health statuses. One participant described how caregiving responsibilities increased significantly after losing their daughter to COVID-19: “I take care of the grandchildren that she left behind. She left behind four kids, and they’re the ones I take care of.” **(Resident Focus Group 1**,** Latiné).** Additionally, primary financial providers falling ill, or having to go without pay to care for another family member added caregiving responsibilities, as another participant stated: “My only provider got sick. He had to be at home. We had to take care of him, support him.” **(Resident Focus Group 1**,** Latiné).**

Participants also discussed the simultaneous responsibility of fulfilling numerous gendered domestic roles and the emotional impact, particularly for women. One resident stated: **“**It was really stressful as a mom and wife because you have to take care of your kids, and you have to take care of your husband, and you have to take care of yourself, too. It was really stressful. It’s something where you say, ‘When is this going to end?’.” **(Resident Focus Group 1**,** Latiné).** Similarly, another resident shared the burden of accumulated challenges while caregiving for their sick husband: “There was also depression, but first, my husband had to live, and he had to stay at home because he had cancer, and he also had lung cancer, so he had to check his bronchial tubes, but there was no place to test, so all the hospitals were closed, so he had to wait. As a mother, I had to take care of my children, but it was so hard to do both of those duties that I relied on God, but it was too vague. It’s too heavy.” **(Resident Interview 1**,** Korean)** Another resident described how children attending school online increased caregiving responsibilities: “It was hard for everyone at that time, the children couldn’t go to school, parents didn’t know how to use computers, and the children didn’t know either. It was hard for everyone.” **(Resident Focus Group 3**,** Latiné).**

#### Navigating service exclusions & inequities

Amid financial strain and increased caregiving demands impacting housing and food stability and taking a toll on physical and emotional wellbeing, residents described navigating various barriers to access basic needs, supports, and essential services such as employment, health insurance, and access to preventive and secondary health care services. Challenges included bureaucratic complexities including insurance requirements, legal status concerns and exclusions, language barriers, lack of paid sick leave, and the digital divide (the gap between those with and those without access to digital technology such as computers, tablets, and smart phones and the internet, often due to socioeconomic factors like income, location, and education). Consequently, many residents found themselves unable to access assistance or unsure of where to turn for help. Several community members brought up unique limitations for undocumented individuals. One participant recounted the inequities faced due to unauthorized legal status: “And in times of the pandemic, like my husband, who was laid off, they didn’t pay him because he didn’t have papers. They just laid him off.” **(Resident Focus Group 1**,** Latiné).** Similarly, others brought up that those without medical insurance faced barriers to accessing care. One participant reflected on the burden of the costs and requirements to access medical help, recounting an example of the significant stress for residents without insurance: “[…] because he didn’t have insurance, he quickly added things up. ‘How much is the ambulance going to cost me?’ He called [me], […] And he, quickly in his imagination, he thought about his health. ‘Come get me because they want to take me in the ambulance, and it’s going to cost me a lot.’” **(Resident Focus Group 4**,** Latiné).** Another resident described the challenge many faced of exposing themselves to COVID-19 risks at work, yet they were neither prioritized for healthcare nor given financial compensation or other benefits if they became ill. This perpetuated a cycle of inequity, as highlighted by the resident: “many healthcare centers prioritized the elderly and the children. But like he said, in some cases, the fathers and mothers weren’t in that age group, but they needed to go to work. They had to take the risk, and by the same token, they couldn’t afford a doctor; and if they stopped working, or they mentioned at work that they had symptoms, they wouldn’t let you work for 2–3 weeks, without getting paid. So, they would lose money, and they had to risk going to work that way, because there was no equal support for everyone regarding healthcare.” **(Resident Focus Group 3**,** Latiné)**.

Lastly, participants often discussed language barriers as compounding COVID-19 inequities and limiting access to services. Participants noted that information sharing was often restricted to English or digital formats, particularly within the diverse Asian American community. One participant described their daily life: “the radius of life was very small” and how **“**[I] didn’t know where to ask for help, I didn’t know how to do it, and I can’t speak English […] I just stayed at home. I came home right after work, and it was a scary time.” **(Resident Interview 2**,** Korean).** Similarly, the digital divide presented a challenge to accessing services, information, and support, as one participant described: “Many students and the parents who had to use technology, nobody knew how to do it. So, stuff like Zoom, or Google Classroom for students, the other one… what’s it called? Google Meets? None of us had ever used any of those things before. So, it was very difficult to learn that.” **(Resident Focus Group 3**,** Latiné).** Another participant described: “And that [using technology] is what our community needs the most help with, especially in Santa Ana, to have more help to learn to use technology.” **(Resident Focus Group 3**,** Latiné).**

### Theme 2: connecting with CHWs

Participants discussed CHW’s comprehensive support. They noted CHWs addressed a wide range of needs, including assistance with navigating worsened and mounting socioeconomic hardships and the resulting emotional toll brought about by the pandemic. Overall, participants expressed a high level of trust and appreciation for CHWs in their community.

#### Engagement with CHW services

Participants described engaging with CHWs as they helped them navigate and access a range of resources and services tailored to their specific needs, such as financial difficulties, housing insecurity, employment issues, food insecurity, Medi-Cal enrollment, and COVID-19 resources and information. Residents described health and finances processes being “really easy” because CHWs provided significant assistance and described CHWs as being “a big help” with navigating Medicare and health finances. When speaking about CHWs who offered them assistance, one resident stated: “for me, she’s an angel” and another said “for me, she’s perfect”.

Some participants emphasized CHWs and CBOs as their primary source for information and resources, as one participant noted: “I have been saying that I rely on the [CBO] for everything. They can solve any problem. Just like that.” **(Resident Focus Group 2**,** Vietnamese)**. Participants discussed examples of CHW support facilitated through various means, including virtual and in-person connections, organizational outreach, phone/text conversations, and on-the-ground information dissemination. One participant described how they sought out CHWs to access resources: “They have excellent CHWs who worked a lot during the pandemic, and I have recently started going there again, because they opened up and you can go in person. So, they have a lot of information for the community, and plenty of assistance too. So, I would go to a CHW to ask for help, where I can get help with my rent, where I can get food. I think they also apply vaccines.” **(Resident Focus Group 3**,** Latiné).** This highlights the agency of community members in seeking assistance from CHWs.

Often participants shared how CHWs and CBOs helped community members become aware of and utilize available resources. They described how CHWs helped connect community members with resources to support paying rent or become informed of their rights against eviction. One resident shared: “But we lost our job. So, we couldn’t make the rent. A lot of people [were] evicted. I was going to be one of them. But […] I went [to CBO]. They helped me.” **(Resident Focus Group 4**,** Latiné).** Residents also shared they became aware of food distribution through the guidance of CHWs and CBOs or received food assistance directly from these entities: “[…] the promoters who also offered us a lot of help. A lot of information. […] they talked to us and told us, ‘Send someone to come and get the food,’ or ‘In such-and-such a place, they’re giving out food. Go there.’” **(Resident Focus Group 1**,** Latiné).** Another participant shared an example of how CHWs assisted with finances and obtaining medical coverage: “Fortunately, when my husband was in the hospital, [the CBO] took care of Medicare […]Even if the hospital bill is high, getting help is a big help. I think it would be really sad if I couldn’t go to the hospital because I had to worry about the cost of the hospital.” **(Resident Interview 1**,** Korean).**

Residents also discussed how CHWs helped them access COVID-19-specific resources and information to protect themselves and others, as one participant shared: “There were agencies, and there were places that gave me masks so that I could wear them when I went outside, to protect myself and protect the people around me.” **(Resident Focus Group 2**,** Vietnamese).** Another participant highlighted the role of CHWs and COVID-19 vaccination: “I also heard from word of mouth about this place. Even though I didn’t want to get vaccinated, but the CHWs were always like, ‘Get vaccinated, get vaccinated, get vaccinated.’ And now, I have all of them (vaccines).” **(Resident Focus Group 1**,** Latiné).**

#### Embracing CHW support and accompaniment

Participants frequently expressed a sense of support from CHWs through regular check-ins and accompaniment navigating complex bureaucratic processes, such as Medi-Cal enrollment and vaccination sign-ups, which residents found overwhelming to handle on their own. Residents described CHWs as “always supporting,” “constantly with me,” and “following up” on their needs. Participants underscored how CHWs’ support fosters a sense of care, emphasizing their thorough follow-up to ensure successful outcomes. One participant noted that when needing to sign up for Medi-Cal, CHWs were instrumental: “[they] were also following up with me, like, ‘If you can’t get it here, I have another form of help. You can go here, or I’ll send you the phone number. Apply for it. Here’s where you can go to apply.’ And if not, they were constantly with me.” (**Resident Focus Group 4**,** Latiné).** Some acknowledged that CHWs had multiple personal and work responsibilities and appreciated the support offered, as highlighted by one participant: **“**Our CHW that’s in our area, […] she makes space, even though she’s got a ton of things to do. Well, for me, she’s perfect.” (**Resident Focus Group 4**,** Latiné)**.

Many participants shared that CHWs offered emotional support, especially during times of isolation and challenges to their emotional wellbeing. These positive interactions highlighted the vital role of community support and how CHWs helped alleviate feelings of loneliness during the pandemic. One participant shared: “[…] when I talked to [CHW], when I started to fill out the rental application, [CHW] said, ‘You’re not alone. We’re here to support you and help you.’ And that was a light that came into my life.” **(Resident Focus Group 04**,** Latiné).** Another participant described CHWs’ regular check-ins: “Because when I had help on the phone, which thank God, I’m never going to stop bringing [CHW] up because she was the one who supported me the most. […] She called me every third day. ‘How are you? How do you feel?’ She was the only one who gave me that resource. And well, what can I say? I mean, she was a great help with that.” **(Resident Focus Group 1**,** Latiné).** Another participant described how CHW virtual check-ins revealed additional areas where CHWs could provide valuable support, noting the CHWs cared for the individual and their support network: “The first thing [CHWs] would ask us, ‘How do you feel?’ And they would watch us. You could say, ‘Fine.’ Uh, ‘And your husband, how’s he doing?’ And they would look for him, like– Because they were watching us. They were looking at us. Then later on, ‘No, well, I have this.’ And then, ‘And what do you need? What can we help you with?’ And one of them would say, ‘Okay, when we finish, I’ll send you a text. We’ll look into it.’ So, that’s what we were looking for– what we had. It was our only option.” (**Resident Focus Group 4**,** Latiné).**

### Theme 3: fostering a community of care

Participants faced the inadequacies of institutional support systems and consequently witnessed the power of community-driven assistance, often facilitated by CHWs and CBOs. Many spoke of the need to “be empathetic” and “be more compassionate,” through shared positive experiences of support.

#### “Pass it on”: the infectiousness of helping

Participants shared a strong sense of supporting each other or “passing it on,” reflecting stories of receiving assistance from CHWs and CBOs, which inspired them to reciprocate. This contributed to a collective wellbeing, establishing a care network necessary for weathering pressing challenges, meeting community needs, and transforming structural injustices. Residents described efforts to coordinate and redistribute resources, including food, transportation, COVID-19 supplies, knowledge, information, and even emotional support. Participants described both relying on CHWs for support and relaying CHWs’ services to others in their community, recounting a shift from individual approaches to collective action. One participant shared how they helped distribute supplies to those unable to get them: “There were people who couldn’t drive, we received the food then shared it with them and shared it with people in that neighborhood. I gave it to anyone who needed it.**” (Resident Focus Group 2**,** Vietnamese)**. Another participant shared how they contributed to alleviating loneliness: “there was someone who was older there in her house alone. I went to spend time with her. I would take her out to walk around the neighborhood as a way to help out.” They also continued to recount helping the community with vaccination: “Also, when the vaccine was out, and they didn’t want it. A lot of people were convinced, and then they got involved. [They said, ] ‘Oh, but I don’t have a ride.’ [and I said] ‘No worries. I’ll take you.’” **(Resident Focus Group 1**,** Latiné).**

One participant exemplified this shift to collective action by describing their journey: “I came to [CHW organization] because of an eviction. And I came looking for help. That’s where I met [CHW]. I stayed there for a while. And then, I saw something that we have to do. We don’t have to complain so much or be the victims but transform our reality. And then, in my case, after an eviction and fighting so hard, I won my case. I had six court hearings, and then, I became a tenant counselor. And from there, I stayed in the community working. Now, I’m an organizer at the center and a tenant counselor. And so, it’s like– we can tell other people, or we can transform ourselves, so we can transform the community.” **(Resident Focus Group 4**,** Latiné).** These experiences highlight the duality of CHWs’ role: addressing residents’ needs where existing systems fall short while fostering a collective care system.

Some participants shared that through engagement with CHWs, they engaged in knowledge building and consciousness-raising to transform their conditions and communities. They shared the knowledge they gained with others, leveraging the power of “word of mouth” to inform their community to advocate for change and maintain the importance of helping one another. Many reflected on the collective strength of organizing. One resident noted “if we organize, we can accomplish a lot of changes.” **(Resident Focus Group 4**,** Latiné)** while another stated “we all need to get involved.” **(Resident Focus Group 4**,** Latiné).** Participants described needing to share what they learned from CHWs. One participant described a need for “Educating myself and training myself to give my service to the community. Because I have the experience of how they supported me and how they guided me. So, I can put what I learned, I can put it towards […] to serve my community.” **(Resident Focus Group 4**,** Latiné).** This sentiment was shared by others, with another participant stating “we have to participate in as a community, and we have to be empathetic to people who don’t know. Now, we know something. We have to pass it on, pass it on to other people, right?”. **(Resident Focus Group 4**,** Latiné)**

#### Attitudes of adaptability

Participants discussed how collective compassion strengthens the community by fostering shared positive experiences of support, which led to positive reflections on community capacity and culture to address challenges. Through collective care, residents demonstrate their ability to strengthen the community’s capacity to recover from crises, maintaining or restoring functionality amid disruptions.

For example, a participant described the strength their community showed, noting, “I know the Vietnamese community very well, that we supported each other, helped each other, gave food to the elderly who were weak, had difficulties and unable to go out. During that time […] I see that our Vietnamese community was very strong [in] that regard” **(Resident Focus Group 2**,** Vietnamese)**. Another participant discussed how the community can take these lessons learned in moving forward. They noted how the community came together to support one another: “It was hard for everyone. So, I think that day by day we can learn and move ahead. But each one of us did the best we could for the community, supporting each other” (**Resident Focus Group 3**,** Latiné).** Many participants noted community organizations, family, and friends as key influences in their ability to adapt. One participant described, “So I have to say that through the pandemic, I can see that the kindness of everyone, the associations, the churches, and the pagodas, all helped the community” **(Resident Focus Group 2**,** Vietnamese).** For many participants, particularly Asian American participants, demonstrating solidarity was strongly linked to a commitment to adhering to public health guidelines. Participants saw this as a way to protect their community, driven by a sense of responsibility for others. One community member emphasized, “In the past, we used to hug and shake hands when greeting, but now we have to take care of hygiene in order to be considerate of others.” **(Resident Interview 3**,** Korean).** Through coping mechanisms and positive reflections about their communities, residents described a sense of unity fostered by overcoming the hardships of the pandemic. This unity may be a key influence towards a desire for more community involvement.

### Theme 4: reinforcing foundations

When discussing their vision for an equitable future, participants described the need to address systemic gaps to improve outcomes for their communities. This often involved active, direct community participation to address urgent needs and future preparedness.

#### Getting involved: “strengths are in our communities”

Participants emphasized the need for community-based solutions that are accessible and tailored to their specific needs. One participant emphasized the need to support and leverage intrinsic community efforts: “The strengths are in our own communities. And it’s just a matter of highlighting them, and putting them into action, and sharing them. Like, we have this instinct. All of us here. I see it. We’ve all been volunteers. We all have that heart, that capacity to give, and to offer, and to serve. So, it’s about empowering ourselves, spreading the word, and always getting involved.” **(Resident Focus Group 1**,** Latiné).** Another participant discussed that in preparing for another crisis “everyone is full of experience” **(Resident Focus Group 2**,** Vietnamese).**

Building on the need to be proactive, one participant stated a need of “not just waiting for [information] to come to us, but to become CHWs, or organizers […]. [To] create a center in our streets, in our neighborhoods. Participate.**” (Resident Focus Group 4**,** Latiné)**. Participants highlighted several areas where the community could get involved, including increasing access to information, training, and resources as well as the necessity for diverse channels of information dissemination to bolster their preparedness for future crises. Participants also expressed a desire to learn more about mental health management, with one participant reflecting that with children going back to school, the community needs to be better prepared “so that we in the community know how to deal with this problem [mental health]. And that’d be great for them to give us that reinforcement and to see.” **(Resident Focus Group 1**,** Latiné)**.

Participants pointed out how organizations need to leverage community members: **“**If we have any information, we will tell everyone, our close ones, our neighbors, our friends. […] word of mouth marketing is effective with Vietnamese people. […] If we just wait for everyone to get the information from the [CBO], it will be slower.” **(Resident Focus Group 2**,** Vietnamese).** In many cases, residents directly connected this need for more information with a call for more CHWs in their communities. One participant described: “The CHWs are very important for our community, but at the same time there are very few of them. Very few people take the risk to go out into the community to knock on doors. I think we need more of that. More CHWs.” **(Resident Focus Group 3**,** Latiné).** Another participant discussed the role CHWs play for those facing barriers to receiving information, including transportation, language, work schedules, lack of access to media, stating “we need more CHWs to visit those people who don’t have transportation, who don’t have the language. They don’t have a way, […] because of their schedule. They work all day. They have their kids. They can’t go out. So, they should come to those houses where there’s no way for people to get that information.” **(Resident Focus Group 1**,** Latiné).**

#### Strengthening social policies

Participants called for stronger policies on economic recovery, healthcare access, sick leave, and food distribution, as well as better access to information and resources. They emphasized the need for institutions to enhance emergency preparedness by expanding CHW models, ensuring non-citizen access to services, and providing multilingual resources.

Participants recurrently expressed the need for fewer barriers to accessing help, including lowering bureaucracy, increasing bilingual staff, and lowering costs or waiting times for assistance. One participant emphasized: **“**I think there should be more flexibility for medical coverage because it’s really expensive to have insurance. […] to qualify for Medi-Cal and all that, there’s a lot of requirements that make it pretty much impossible.” **(Resident Focus Group 1**,** Latiné)**. Reflecting the need for fewer barriers, another participant shared: “they gave you the help that they gave you, sure. But they gave it to you, like, ‘I’ll give it to you,’ but they took it back. Because they put requirements, […] if they’re going to give something, they should give it. Don’t put up so many barriers, so much information, or so many things so that you can apply.” **(Resident Focus Group 1**,** Latiné).** This underscores the need for policies that address barriers to access, as availability alone does not guarantee utilization.

Immigration status frequently emerged as a barrier to access, with many concerns about potential repercussions or being labeled a public charge. One participant underscored the need for more inclusive services: “We need more centers to help the community, where you can go there as a person. You won’t go to social services to ask for anything. Why? Because you know that they seldom give anything to undocumented people. But if you go to a community center […], where your migration status doesn’t matter, then you go there with confidence and you can say, ‘I need to pay my rent, can I get some help with that?’ Then you would qualify without the fear of being charged for it later on, or that they will deport you or something like that. […] So, there are many reasons why people in the community won’t ask for help. But there is also the fear of not knowing what will happen to their migration status.” (**Resident Focus Group 3**,** Latiné).**

Community members expressed not always being aware of available services. As one participant shared: “[It] would be a really good form of help if there were more CHWs, so they could do this function of informing all the people. […] Sometimes, while you’re chatting at the laundromat or at the store, you realize that there’s all these resources. And you’re like, ‘Where? When? Why didn’t I know about it? I missed it, and I wasn’t there.’ So, that kind of information that escapes you and that’s out there.” **(Resident Focus Group 1**,** Latiné)**. Some stressed the need for information in their native language. As one participant shared: “I think we need to get a lot of information. […] it always comes in English, but it’s hard because it doesn’t come in Korean.” **(Resident Interview 2**,** Korean).** The need for training in computer usage, internet navigation, and communication platforms like Zoom, which became essential yet under-addressed needs in the community was also highlighted. One participant shared: “[…] there should be more workshops for parents and for the whole community, […] regarding technology. Free classes. I know there are some, but they should promote them more. They should be held in spaces for the community, closer to the people, and to advertise them” **(Resident Focus Group 3**,** Latiné)**.

## Discussion

This study provides valuable insights into the breadth and depth of community members’ experiences and responses during the COVID-19 pandemic. It highlights the accumulated and compounding issues that low-income, immigrant communities face, the key role of CHWs in both the immediate response and long-term recovery, and the need to support community efforts that are integral in attenuating these challenges. Our findings align with the multisystemic promotores framework [15], wherein CHWs work to meet direct social and health needs, drive systems change through community mobilization, and drive capacity-building within the community.

### Compounding interconnected challenges require holistic CHW support

The complexity and interconnectedness of the pandemic-related challenges communities faced underscore the importance of understanding community needs and identifying necessary resources for support and wellbeing [40]. It also highlights the need for programs that address multiple overlapping needs, recognizing that disruptive events rarely affect just one aspect of socioeconomic stability and overall wellbeing [41]. Through their efforts, CHWs provided comprehensive support to help residents navigate the compounded challenges they faced, filling critical gaps in the governmental response and enabling access to public services [12]. While this holistic role gained prominence during the COVID-19 pandemic, CHWs have long been recognized for addressing broader determinants of health such as housing, food security, and emotional wellbeing [8]. It is important that these efforts be supported and sustained, through for example, secure funding, flexible reimbursements mechanisms, and proper recognition of the workforce [42, 43].

### Connecting with CHWs leads to a community of care

CHW and CBO efforts significantly contribute to alternate community-driven responses to inequities, grounded in an understanding that those facing crises possess the best knowledge for solutions and abilities to collectively drive change [18]. As such, CHW efforts have a chain reaction grounded in an environment of mutual support that shapes COVID-19 recovery through a collective sense of care and mutuality [44]. The CHW model inspired residents to support, accompany, and share resources with neighbors and other community members to address unmet needs and mitigate community-level injustices, and cultivate characteristics of CHWs (e.g., emotional support) in their own communities [18]. This highlights how the CHW role is essential in short-term efforts and in long-term recovery. Furthermore, this model helps in creating a continuum of engagement that CBOs can leverage when hiring experienced community leaders [45].

### Reinforcing foundations through community power

Findings showed how already impacted communities supported themselves during the pandemic, reiterating their capacity and knowledge for recovery. Globally, evidence of community-led mutual aid showed individuals sharing resources and care, especially with those at higher risk [46]. Recognizing these initiatives and developing strategies to sustain community-led initiatives is essential for long-term equity [47]. Communities recognize their collective strength and acknowledge that foundational systems are broken. To address structural determinants of health, it is essential to shift power relationships among socially constructed groups to disrupt cycles of advantage and inequity [48]. By transforming the dynamics that drive these inequities, communities can lead in building stronger local economies and promoting collective wellbeing, focused on creating upward cycling patterns for sustained growth and addressing the root causes of inequities. Cultivating various forms of capital, including social, economic, cultural, and symbolic, is crucial for strengthening communities and fostering mutual aid during disasters [47]. Our findings highlight the role of CHWs in community building and strengthening health. In extending these recommendations, LeBrón and colleagues (2024) [16] lay out a CHW-led vision for neighborhood-based ecosystems of health, wherein CHWs support community-building for transforming social relationships, physical spaces, and social policies to promote health.

### Limitations/Strengths

A key strength of this study is its focus on the perspectives of community residents, centering their experiences during the COVID-19 pandemic and recovery, thus providing valuable underrepresented insights. The qualitative approach yielded rich, contextual data, and the sample size was adequate to ensure comprehensive representation of community experiences. The study is further enhanced by the implementation of a CSW model, which builds on long-standing community-academic collaborations. CSWs brought valuable community knowledge and skills in relationship-building, facilitating discussions, and sensitively probing when appropriate. This encouraged open conversations with residents who might have been reluctant to share their experiences with academic researchers. By employing a community-based participatory research approach, the study not only strengthened community capacity but also yielded practical research implications and enhanced the trustworthiness of the data.

Nonetheless, our study isn’t without limitations. While the COVID-19 pandemic significantly impacted Native American and Black communities in Orange County, our study did not include residents from these groups. Instead, our data provides a deeper understanding of the experiences of Asian American and Latiné communities. Additionally, the time lapse between the pandemic’s onset in early 2020 and our interviews (2023) may have introduced recall bias, potentially leading to less specific recollections of the pandemic’s initial stages. A consequence of which might be under-reporting of the severity of COVID-19 experiences and potentially forgetting about community responses during that period.

### Recommendations/Implications for policy and practice

It is imperative to support and enable communities in continuing their crucial work. Adequate funding for CHWs and CHW organizations is essential, as their structural and cultural knowledge and community trust allow them to strengthen communities through multisystemic approaches. Communities must be allowed to inform policies and plans by actively listening to communities during the development of interventions and solutions. Our findings indicate that residents call for an increased presence of resources, greater community involvement, and better promotion of services. Additionally, sustainable policies for future crises must prioritize community needs, recognizing that public health crises like COVID-19 affect everyone. These policies should emphasize inclusivity for traditionally excluded groups and ensure support for diverse job types, paid leave, and accessible healthcare for all.

## Conclusions

This qualitative study sought to understand the experiences of low-income, immigrant communities of color with CHWs during the pandemic. This study applied an innovative process of engaging CHWs in each phase of the research process as Community Science Workers. Findings indicate the profound interconnected socioeconomic, health, and caregiving challenges worsened or brought on by the pandemic; the essential role of CHWs in connecting with and accompanying residents to address multi-layered unmet needs; the spillover effects of CHW supports to resident-led community of care; and resident recommendations to address systemic gaps to support community rebuilding and to strengthen preparedness for future public health crises. Sustaining and growing CHW models and authentically engaging residents in processes that determine community resources and the allocation of resources in ways that center historically and contemporarily marginalized communities, informed by lessons from effective CHW models, hold promise for improving community health and health equity.

## Data Availability

The datasets used and/or analyzed during the current study are available from the corresponding author on reasonable request.

## Abbreviations

CBO: Community based organization
CHW: Community health worker
COVID-19: Coronavirus disease 2019
CSW: Community science worker
ICA: Inter-Coder agreement
